# Hormones removal from municipal wastewater using ultrasound

**DOI:** 10.1186/s13568-018-0621-4

**Published:** 2018-06-01

**Authors:** Aliakbar Roudbari, Mashallah Rezakazemi

**Affiliations:** 1 Center for Social and Behavioral Sciences Research, Shahroud University of Medical Sciences, Shahroud, Iran; 2 Faculty of Chemical and Materials Engineering, Shahrood University of Technology, Shahrood, Iran

**Keywords:** Estrogens, Ultrasound, Wastewater, Reduction, Hormones

## Abstract

Estrogens are one of the micro-pollutants in the wastewater which have detrimental effects on water living organisms. The aim of this study was to evaluate the efficiency of ultrasound to reduce the estrogen (E1) and 17 beta-estradiol (E2) from municipal wastewater. Hence, a cylindrical batch reactor was designed. The effects of powers, frequency, exposure time and pH on reduction efficiency were investigated. The residual concentration of E1 and E2 hormones was measured in reactor effluent by electrochemiluminescence (ECL) method. The results showed that ultrasound removed 85–96% of both E1 and E2 hormones after 45 min while other parameters changes in the range of their operations. Also, the frequency and power of ultrasound had a significant effect on reduction efficiency of hormones while the exposure had no significant effect. Furthermore, the interaction of power and frequency reduced their efficacy to 64.3% (P_value_ = 0.005). The result also indicated that the ultrasound waves have high ability to reduce Steroid hormones from municipal wastewater. The proposed method can be considered as one of the significant strategies for reduction or destruction of hormones from wastewater due to the non-generation of dangerous by-products and the low energy consumption.

## Introduction

Estrogens are one of the micro-pollutants in wastewater which have detrimental effects on water living organisms (Azimi et al. 2017; Hamid and Eskicioglu 2012). These hormones are divided into five types: Progestin (Progesterone), Glucocorticosteroids (Cortisol), Mineral corticosteroids (Aldosterone), Androgen (Testosterone), and Estrogen (Nagarnaik et al. 2010). Estrone (E1) and 17 beta-estradiol (E2) are the most important estrogen hormones in wastewater which are excreted by all humans and animals (Guedes-Alonso et al. 2014). Estriol (E3) and 17-alpha-ethinyl estradiol (E4) are other estrogens which are found in smaller amounts in wastewater (Blair et al. 2013). The existence of these compounds in wastewater was firstly reported in 1965 (Hamid and Eskicioglu 2012) but was not seriously investigated by researchers until 1980 that the detrimental impacts of these hormones were confirmed on fish growth (Behera et al. 2011). Steroid hormones are endocrine-disrupting compounds in the body which have become one of the major concerns as wastewater effluent in the environment because of negative effects on human health, animals, and ecosystem balance (Aker et al. 2016; Mendoza et al. 2016). Estrogen in very low concentrations (less than 0.1 ng/L) interferes with reproduction of human, livestock, and wildlife and has a stimulatory effect on breast tumor growth (Ravindran et al. 2016). Some studies have shown its effect on uterine cancer, ovary and other cancers (Yi et al. 2016). These hormones either are produced naturally in the human and animal body or are found in some materials that humans deal with them on a daily basis. Detergents, shampoos, lotions, and cosmetics are new sources of these hormones in environment and wastewater.

The levels of estrogens are increasing in municipal and industrial wastewater due to increasing usage of these compounds (Cedat et al. 2016; González et al. 2015). Increasing the level of these substances in water resources and wastewater led to increasing attention of researchers and national and regional authorities to them and several studies have been conducted to determine their concentration in liquid environments. In a study in Brazil in 2012, E1 and E2 concentrations in raw wastewater were determined 566 and 143 ng/L, respectively (Pessoa et al. 2014). Also, in the study on wastewater treatment lagoons in the US in 2011, the concentration of these hormones was reported 16.9 and 126 ng/L, respectively (Luo et al. 2017). Also, the study conducted in South Korea in 2004 indicated E1 hormone levels in surface waters at 1 ng/L (Kim et al. 2007). Some of the studies on hormones are attributed to how to remove or decrease the amount of them in water resources and wastewater. Different methods are used in these studies; the most important methods are: activated sludge along with biological nutrient reduction (BNR) (Phillips et al. 2016) without reduction of biological nutrients (nBNR) (Sornalingam et al. 2016), oxidation ditch (Li et al. 2013), aeration lagoon (Li and Ni 2011), the combination of nanofiltration and reverse osmosis (Plósz et al. 2010), activated carbon adsorption (Foroutan et al. 2017; Furgal et al. 2015), peroxon process (Zhang et al. 2015), water chlorination and photo-Fenton-like degradation (Ifelebuegu et al. 2016). Wastewater treatment has been the subject of several recent studies (Baheri et al. 2014; Rezakazemi et al. 2018b; Muhammad et al. 2017; Rezakazemi 2018; Rezakazemi et al. 2011a, b, 2012, 2013, 2014; Rezakazemi et al. 2018a, c; Shahverdi et al. 2013; Shirazian et al. 2012). Indeed, the efficiency of these methods for reducing the hormones is different and some of them were unable to provide acceptable reduction efficacy (Wojnarowicz et al. 2014). According to studies, biological treatment methods and advanced treatment processes have better ability to remove or reduce the amount of hormones. Although biological treatment can eliminate hormones, some hormones remain in the effluent (Auriol et al. 2008). Advanced treatment methods have higher ability to remove hormone, but they are faced with two limitations of high costs and dangerous by-products formation (Moreira et al. 2017).

In general, most of the methods proposed for the removal of hormones are on a laboratory scale and there are few real scale examples. One of the most effective methods to remove hormones without by-products generation is ultrasound (waves with a frequency greater than 20 kHz). Ultrasound waves which had discovered by Francis Galton in 1876 are produced by two methods: Piezoelectricity (interaction of mechanical pressure and electrical power), and Magnetostriction (Generation of ultrasonic waves in the electromagnetic field) (Musielak et al. 2016). This method is used for remedy of new injuries and old and chronic inflammations, recovery of elastic skin and reduction of organic chemical pollutants from liquid environments (Mahravan et al. 2016).

The aim of this study was to investigate the effect of ultrasound on the reduction of sewage hormones from wastewater and not effluent. To achieve this purpose, samples were taken from the end of wastewater collection network before entering the first unit of the treatment plant. Investigations showed that very few studies have been done on the effects of ultrasound on reducing hormones parameters such as the effect of power and different frequencies, the effect of exposure time to ultrasound, the effect of initial pH, and also the interaction of ultrasound frequency and power. Therefore, the aim of this study was to investigate the effect of power, frequency, exposure time, initial pH, and also the interaction of ultrasound frequency and power on reduction efficiency of Estrone and 17 beta-Estradiol from municipal wastewater. Moreover, the main aspects of this technology have not been studied yet.

## Materials and methods

### Chemicals

All required chemicals including ferrous sulfate, sulfuric acid, and hydrogen peroxide were purchased from Sigma-Aldrich. In this study, E1 and E2 hormones were studied. The existence of these hormones in wastewater with higher concentrations than other hormones, as well as differences in chemical structure, molecular weight, and their properties were the reasons for choosing these two hormones. The concentrations of these hormones in wastewater were between 485 and 535 ng/L. Sulfuric acid was used to adjust pH. Ferrous sulfate and hydrogen peroxide were used for sample preparation in ECL method.

### Wastewater characteristics and preliminary experiments

Wastewater used in this study was prepared from Shahrood wastewater treatment plant. This treatment plant uses stabilization ponds process and is the largest treatment plant in Semnan province. Samples were taken from the entry of the first pond. The characteristics of wastewater are described in Table 1. Samples after collecting were tested immediately to minimize biochemical changes. The amount of nitrogen ammonia and pH were measured by C203 8 parameter test meter device (Hanna Electronics Company) and benchtop pH meters (Cole-Parmer Co., Ltd Company). The pH meter was calibrated before each use with pH 3, 7 and 10 buffer solutions. The biochemical oxygen demand (BOD) and chemical oxygen demand (COD) measurements were determined following Standard Methods 5210 and 5220, respectively. Total solids (TS) were measured through evaporation in a furnace at 105 °C for 1 h.

**Table 1:** Characteristics of wastewater

Parameter	Value	Unit	Parameter	Value	UNIT
Total solids	1150 ± 65	mg/L	TOC	156 ± 16	mg/L
COD	340 ± 48	mg/L	pH	7.2 ± 0.35	–
BOD	225 ± 43	mg/L	Ammonia nitrogen	652 ± 34	mg/L
E1	485 ± 32	ng/L	E2	511 ± 16	ng/L

### Reactor characteristics

**Fig. 1:**
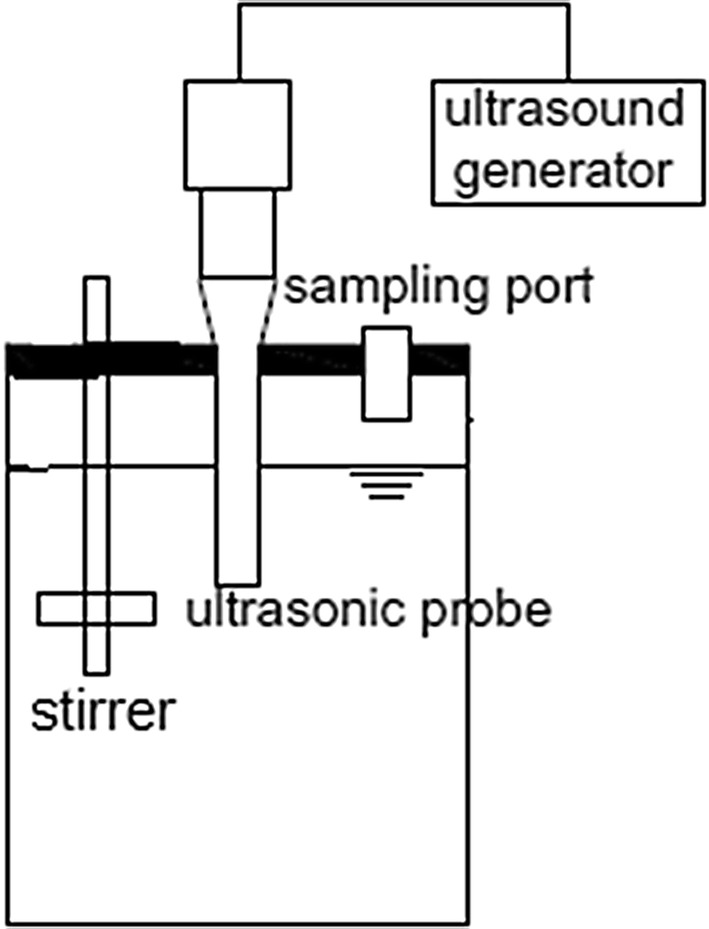
Schematic of reactor

In this study, a cylindrical reactor made of Plexiglas in the amount of 1.5 L for the batch reactor was designed (Fig. 1). The reactor contents were stirred by a stirrer magnet with low speed (450 rpm). The source of ultrasound generation was the device Model UGMA-5000 (Sonotek Company) with three transducers 30, 45 and 60 kHz equipped with a titanium probe with a diameter of 20 mm, operated over a frequency range of 25–250 kHz with a display resolution of 0.01 Watts and ± 4% accuracy leading for increased repeatability. The probe submerging depth in the reactor was 22.5 cm (half of the reactor depth).

### Experimental setup

In this study, the effect of ultrasound power, frequency and exposure time and also the initial pH of wastewater on the reduction of E1 and E2 hormones were investigated. For this purpose, the effect of powers (70 and 110 W), frequency (30, 45 and 60 kHz), exposure time (30, 60, 90 and 120 min) and pH (3, 7 and 10) were investigated. Also, each test was repeated 3 times, and residual concentration of E1 and E2 hormones was measured in the reactor effluent.

### Analytical method

Hormone concentration was measured by electroluminescence (ECL) method (Kische et al. 2016). For this purpose, the joint product of Roche and Hitachi, Elecsys 2010, was used. In this method, 35 μL of the sample with a specific estradiol antibody constitute an immune complex. Then the tiny spherical constituents coated with streptavidin are added to the environment that consequently, places of antibodies that are still empty can form an antibody mixture (Hapten) and are filled with it. Then, all mixture transferred to a solid phase during an interaction and being pulled into measuring cell. In this cell, micron particles magnetically trapped on the electrode surface and substances that have not been trapped will be removed by Procell. Then voltage is added to the electrode to cause emission of chemiluminescence light. The amount of the light is measured by a light multiplier. Then, the result is taken to the calibration curve and the amount of hormones was determined (Cacho et al. 2016). A detection limit of ECL was adjusted to 1–500 ng/L.

### Statistical analysis

In this study, the effects of frequency, power, exposure time, and initial pH were evaluated on the amount of the E1 and E2 reduction from municipal wastewater. SPSS version 19 was used for statistical analysis. One way ANOVA was used for evaluating the effect of frequency, exposure time and pH and Independent Sample *T* Test was used for evaluating the effect of power. Tukey HSD was used to determine which value of the variable was effective to remove hormones. Univariate analysis was used to evaluate the effect of interaction and Levene test is used to determine the equal or unequal variances in different groups for each variable. When the variances in different groups were not being equal, Kruskal–Wallis test (its nonparametric equivalent) and Mann–Whitney test were used instead of one-way ANOVA and independent sample *T*-Test, respectively.

## Results

**Fig. 2:**
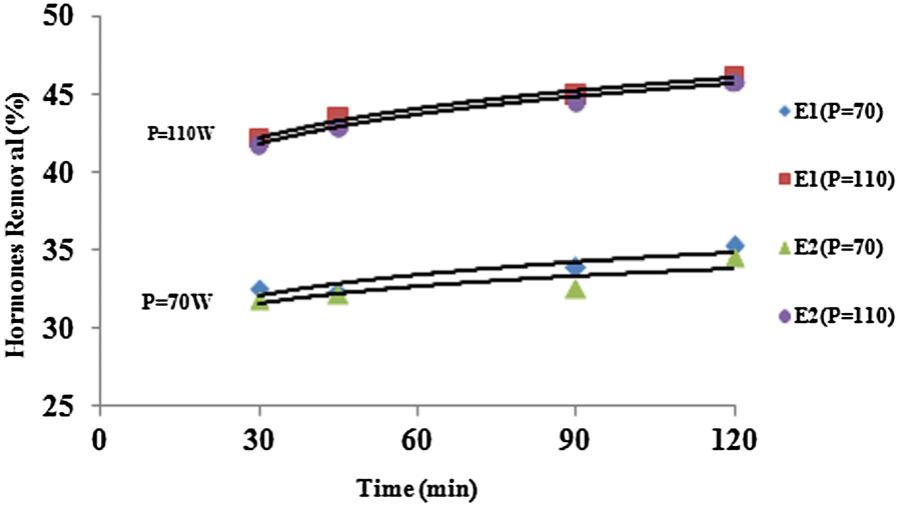
Effect of power on E1 and E2 reduction (frequency = 45 kHz, pH 7)

**Fig. 3:**
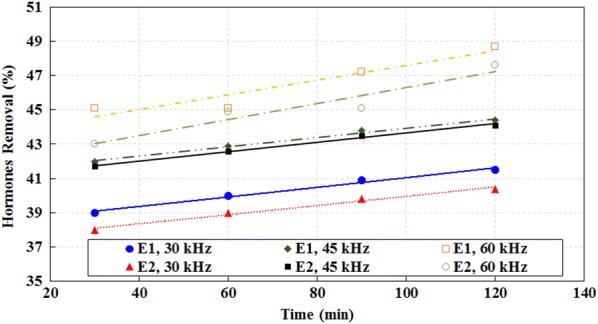
Effect of frequency on E1 and E2 reduction (power = 70, pH 7)

**Fig. 4:**
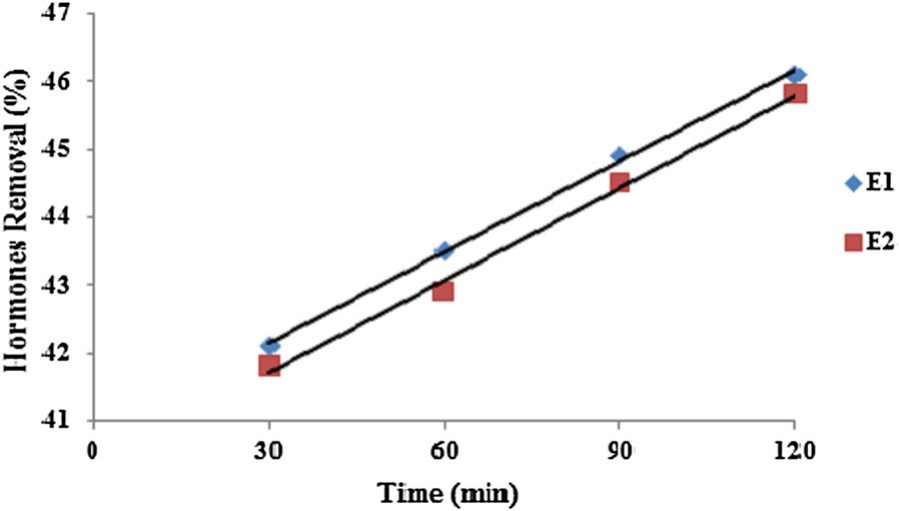
Effect of exposure time on E1 and E2 reduction (power = 110, frequency = 60 kHz)

**Fig. 5:**
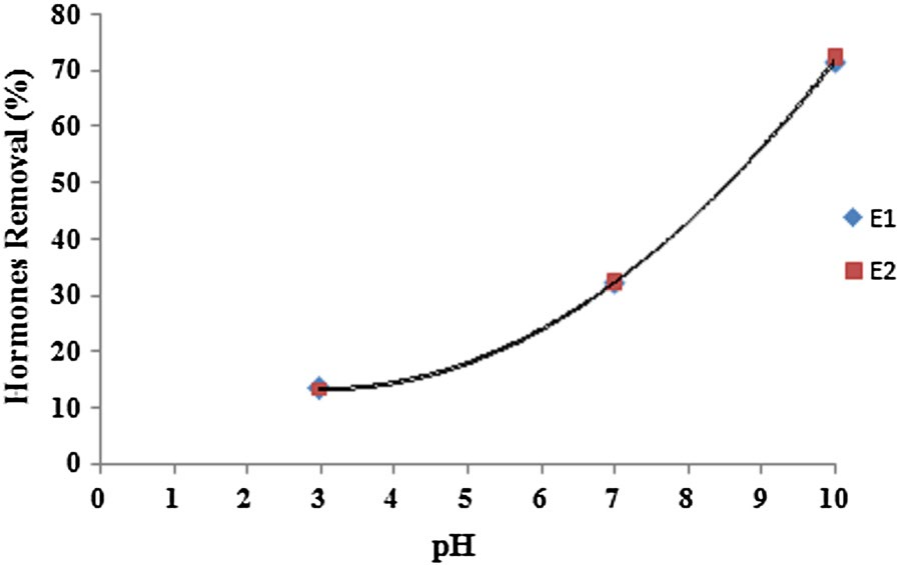
Effect of pH on E1 and E2 reduction (power = 110, frequency = 60 kHz)

Table 1 shows the wastewater characteristics which were used in the study. As can be seen, the wastewater was clearly representative of municipal wastewaters. The studies performed by Sun et al. (2016) and Renuka et al. (2016) showed similar results. The results showed that ultrasound waves had the high ability on reducing hormones E1 and E2 (Tables 2 and 3). Figure 2 shows the effect of power on E1 and E2 reduction. As can be seen, with increasing the ultrasound power increases the efficacy reduction of E1 and E2. Figure 3 shows the effect of frequency on E1 and E2 reduction. As can be seen, with increasing ultrasound frequency, the reduction efficacy of E1 and E2 has increased. Figure 4 shows the effect of exposure time on E1 and E2 reduction. As can be seen, with increasing exposure time, the reduction efficacy of E1 and E2 somewhat has increased but the not significant. Figure 5 shows the effect of pH on E1 and E2 reduction. As can be seen, with increasing pH, the reduction efficacy of E1 and E2 has increased.

**Table 2:** Reduction rate (%) of estrogen by ultrasound at different time, power, pH and frequency (Mean ± SD)

pH	Power (W)	Frequency (KHz)	Time (min)			
			30	60	90	120
3	70	30	12.4 ± 2.3	12.7 ± 1.4	13.1 ± 1.3	13.2 ± 1.3
3	70	45	13.3 ± 1.8	13.5 ± 1.6	13.8 ± 1.2	14.5 ± 1.2
3	70	60	14.3 ± 1.3	15.6 ± 1.4	15.8 ± 1.2	16.5 ± 1.3
3	110	30	20.5 ± 2.4	21.1 ± 2.1	21.5 ± 1.7	21.9 ± 1.6
3	110	45	22.3 ± 1.5	22.7 ± 1.5	22.9 ± 1.4	23.3 ± 1.1
3	110	60	23.5 ± 1.2	23.7 ± 1.2	24.1 ± 1.4	24.3 ± 1.2
7	70	30	26.3 ± 1.4	26.7 ± 1.2	29.6 ± 1.5	31.2 ± 1.4
7	70	45	32.5 ± 1.2	32.3 ± 1.1	33.8 ± 1.3	35.2 ± 1.6
7	70	60	31.5 ± 1.3	33.1 ± 1.2	35.6 ± 1.2	38.9 ± 1.8
7	110	30	39.1 ± 2.5	40.5 ± 2.9	40.8 ± 2.3	42.3 ± 3.1
7	110	45	42.1 ± 3.2	43.5 ± 2.7	44.9 ± 2.6	46.1 ± 2.5
7	110	60	46.0 ± 2.5	46.1 ± 2.8	47.8 ± 2.6	49.2 ± 2.5
10	70	30	67.1 ± 3.1	68.9 ± 3.6	69.5 ± 2.7	70.6 ± 2.8
10	70	45	70.8 ± 2.6	71.5 ± 2.5	74.2 ± 2.7	75.3 ± 2.6
10	70	60	75.6 ± 2.5	75.9 ± 2.6	77.5 ± 2.8	79.6 ± 2.6
10	110	30	81.3 ± 2.6	81.9 ± 2.5	82.6 ± 3.1	84.6 ± 3.1
10	110	45	85.8 ± 3.7	86.9 ± 2.6	87.2 ± 3.5	90.9 ± 3.2
10	110	60	91.1 ± 3.1	91.2 ± 3.1	92.2 ± 3.2	94.2 ± 2.7

**Table 3:** Reduction rate (%) of 17 beta-estradiol by ultrasound at different time, power, pH and frequency (Mean ± SD)

pH	Power (W)	Frequency (KHz)	Time (min)			
			30	60	90	120
3	70	30	12.2 ± 1.8	12.5 ± 1.6	13.0 ± 1.1	13.1 ± 1.3
3	70	45	13.1 ± 1.2	13.2 ± 1.3	13.3 ± 1.2	13.7 ± 1.4
3	70	60	13.8 ± 1.1	14.5 ± 1.2	15.1 ± 1.2	15.8 ± 1.1
3	110	30	19.8 ± 1.3	21.0 ± 1.3	21.2 ± 1.2	21.6 ± 1.4
3	110	45	21.9 ± 1.3	22.3 ± 1.2	22.5 ± 1.4	23.1 ± 1.4
3	110	60	23.1 ± 1.4	23.5 ± 1.4	24.0 ± 1.3	24.1 ± 1.4
7	70	30	25.8 ± 1.6	26.4 ± 1.5	28.8 ± 1.5	30.8 ± 1.3
7	70	45	31.8 ± 1.2	32.1 ± 1.3	32.5 ± 1.2	34.5 ± 1.2
7	70	60	30.8 ± 1.3	32.5 ± 1.2	34.2 ± 1.4	37.5 ± 1.6
7	110	30	38.6 ± 1.3	39.8 ± 1.2	40.5 ± 1.2	41.8 ± 1.4
7	110	45	41.8 ± 1.3	42.9 ± 1.4	44.5 ± 1.3	45.8 ± 1.4
7	110	60	43.0 ± 1.2	45.6 ± 1.4	46.2 ± 1.3	48.2 ± 1.5
10	70	30	66.5 ± 2.3	67.8 ± 2.1	69.2 ± 2.2	70.8 ± 2.3
10	70	45	70.9 ± 2.4	72.3 ± 2.2	74.2 ± 2.3	75.5 ± 2.1
10	70	60	75.3 ± 2.2	75.9 ± 2.1	76.8 ± 2.3	78.5 ± 2.1
10	110	30	81.9 ± 2.4	82.3 ± 2.3	82.5 ± 2.8	83.9 ± 2.6
10	110	45	86.2 ± 2.5	86.9 ± 2.6	86.6 ± 2.8	88.9 ± 2.6
10	110	60	90.9 ± 2.4	91.3 ± 2.5	91.8 ± 2.3	93.6 ± 2.2

## Discussion

In this study, the effect of power, frequency, exposure time, initial pH, and also the concurrent effect of power and frequency on Estrone and 17 beta-Estradiol reduction from municipal wastewater were investigated. According to the results, ultrasound can reduce 85–96% of E1 and E2 after 45 min while other parameters changes in the range of their operations and ultrasound frequency and power had a significant effect on reduction efficiency but exposure time had no significant effect. Also despite the fact that the power and frequency individually had a significant effect on hormones reduction, but their concurrent effect was reductive. With the increasing initial pH, reduction efficacy increased due to the increased production of hydroxyl radicals. The result indicated that the ultrasound has high ability to remove Steroid hormones from municipal wastewater, so due to non-generation of dangerous by-products and low electricity requirement; this method can be considered as a valuable strategy for reduction or destruction of hormones from water resources.

The effect of frequency and power on reducing E1 and E2 was significant but the effect of exposure time was not significant. Statistical analysis of Univariate showed that the power individually had effective rates of 89.9 and 90.1% on E1 and E2 reduction, respectively, and the frequency individually had effective rates of 80.1 and 82.2% on E1 and E2 reduction, respectively. But the concurrent effect of frequency and power was significant (P_value_ for E1 and E2 were 0.008 and 0.006, respectively) and decreases (reduction efficacy for E1 and E2 were 68.5 and 70.1%, respectively).

In fact, ultrasound waves lead to extensive destruction of organic materials, particularly macromolecules and massive organic materials by the formation of hydroxyl radical and cavitations phenomenon (Budiman and Wu 2016; Wu et al. 2012). E1 and E2 hormones have strong oxidizing properties, so they are easily oxidized by hydroxyl free radicals produced by ultrasound (Renuka et al. 2016). Also, these compounds are exposed to cavitation (Hotspot) made by ultrasound and they will remove because they have carbon ring of cyclohexane and cyclopentane types and desirable volatility properties (Jia et al. 2015). Andaluri et al. (2012) in a study showed that ultrasound can destruct some hormones through the production of hydroxyl radical and cavitation phenomenon.

According to Sono-chemical theory, when the ultrasound power increases more than the cavitations threshold, bubbles are formed faster and more and release its energy easily (Andaluri et al. 2012), so a large number of cavitations bubbles explode with high energy in a short time and destroy hormonal molecules attached to themselves, thus the efficiency of hormones reduction increases. Statistical analysis of independent sample T-Test showed that there is a significant difference between E1 and E2 concentrations in different powers in reactor influent and effluent (P_value_ = 0.001 for both hormones). The results of the study are consistent with the study of Andaluri et al. (2012). They studied ultrasonic oxidation of diethyl phthalate and concluded that with increasing the power, the removal efficacy increases.

The increasing of frequency leads to increasing the number of cavitations bubbles and also the number of hydroxyl radicals in the environment (Suri et al. 2007), so the reduction efficacy of E1 and E2 increases. The statistical analysis of one way ANOVA showed that there is a significant difference between E1 and E2 concentrations in different frequencies in reactor influent and effluent (P_value_ for E1 and E2 are 0.001 and 0.002 respectively). Also, statistical analysis of Tukey showed there is a significant difference between 30 and 60 kHz frequencies (P_value_ for E1 and E2 are 0.001 and 0.002, respectively) and between 45 and 60 kHz frequencies (P_value_ for E1 and E2 are 0.002 and 0.001, respectively) on E1 and E2 reduction. Suri et al. (2007) confirmed the effect of frequency on hormones reduction in their study.

In fact, because the formation of hydroxyl radical and cavitations bubbles begin to operate in very short time, hence with increasing exposure time, the reduction efficiency will increase slightly (Selvaraj et al. 2015), in another word the majority of these hormones are removed in early times. Also, the statistical analysis of one-way ANOVA showed there is no significant difference between E1 and E2 concentrations in reactor influent and effluent at different exposure times (P_value_ for E1 and E2 are 0.17 and 0.21, respectively).

With increasing pH, the number of hydroxyl radicals produced in the environment is increased (Okada et al. 2009) and consequently the rate of oxidation and hormone reduction increases, so as can be seen from Fig. 5 the alkaline environment increases hormones reduction. Statistical analysis of one way ANOVA showed there is a significant difference between E1 and E2 concentrations in reactor influent and effluent at different pH (P_value_ for E1 and E2 are 0.17 and 0.21, respectively).
